# Tracing the evolution of health policymaking in Saudi Arabia: a qualitative analysis of experts' opinion

**DOI:** 10.3389/fpubh.2025.1672410

**Published:** 2026-01-22

**Authors:** Bayan A. Hariri, Faisal M. Albagmi, Afnan A. Aljaffary

**Affiliations:** 1 Health Information Management and Technology Department, College of Public Health, Imam Abdulrahman bin Faisal University, Dammam, Saudi Arabia; 2 Physical Therapy Department, College of Applied Medical Sciences, Imam Abdulrahman Bin Faisal University, Dammam, Saudi Arabia

**Keywords:** health policy reform, policy evolution, policy implementation, qualitative research, Saudi Arabia

## Abstract

**Background:**

A combination of international policy adoption and local implementation strategies has historically shaped health policy development in Saudi Arabia. However, there is limited understanding of the factors driving these policy changes and the challenges encountered during implementation.

**Objectives:**

This study aims to explore the state of health policymaking in Saudi Arabia, how it is formulated, and how it has evolved over the years.

**Methods:**

This qualitative study used thematic analysis of expert interviews to identify key policy formulation and evolution themes. It analyzed expert insights from 10 semi-structured interviews with policymakers, thematically coded the data, and examined it using NVivo to uncover patterns in health policy evolution.

**Results:**

The findings indicate a shift from centralized policymaking to a more collaborative, evidence-based process. Interviewees highlighted various challenges in policy implementation, including a lack of policy governance and an insufficient policy evaluation process.

**Conclusion:**

This study underscores the importance of strengthening local policy capacity to reduce dependence on foreign experts and ensure that policies align with national needs. Additionally, establishing a centralized policy repository would improve transparency, while investing in a robust health data infrastructure would bolster evidence-based policymaking and adaptive health reforms.

## 1 Introduction

Public health policies comprise a set of decisions, plans, and actions made by governments to address healthcare-related issues and guide the overall direction of the healthcare system. They cover various areas, including access to healthcare delivery models, healthcare services, and public health initiatives and regulations (1). They are typically developed through a complex process involving research, healthcare needs analysis, stakeholder consultation, and consideration of social, economic, and political factors (2). They are crucial because they are formulated and implemented by the government at the macro level, addressing objectives that have a widespread influence on the entire population (1).

Historically, the course of public health policymaking has shifted notably from a reactive, infrastructure-focused approach that concentrated on tackling outbreaks such as malaria and plague and/or building basic infrastructure for health services delivery to a more proactive, holistic one that emphasizes population health (3). This shift highlights a key point: public health policymaking is not just a series of procedures but involves a meaningful redefinition of the field itself, evolving from a narrow, reactive fight against contagion to a broad, proactive effort that considers the complex social and behavioral factors influencing population health (4). Although this has been noted globally, health policymaking differs across countries and regions due to variations in healthcare systems, political contexts, cultural influences, societal values, and geographic epidemiology (2).

Despite these differences, policymakers, those who “contribute to, influence, or directly involved in making policies,” universally undertake a pivotal role in shaping the direction of healthcare reforms, overseeing their implementation, and evaluating their impact on various dimensions of the healthcare landscape (4). Through their decision-making authority, policymakers shape resource allocation, regulatory frameworks, and governance structures, ultimately influencing healthcare accessibility, quality, and effectiveness (1).

At the country level, Saudi Arabia is undergoing a significant transformation in its health sector, accompanied by the implementation of numerous macro-level policies. In 2016, the Health Sector Transformation Plan (HSTP) was launched following the inception of Vision 2030. This plan aims to restructure the health sector by reforming the funding, delivery, and regulating health services. This was achieved by introducing policies to enhance efficiency, improve access to care, and better utilize financial resources. Consequently, the role of the MoH focused on regulating the sector, while funding and delivery were transferred to other entities (5). The HSTP initiated a significant restructuring of the Saudi health sector, fundamentally changing the dynamics of health policymaking.

Research findings have shown that although this nationwide transformation has made significant changes, it still faces numerous challenges at various levels. Data revealed shortcomings in research evaluating the progress of these reforms and how they can inform policies (5–7). For example, there is a considerable need for research in areas such as Public-Private Partnerships (PPPs), which is a key enabler of the privatization movement that the reforms promote, as well as research in other reimbursement models, especially with the introduction of Value-based Healthcare (VBHC) (5). Other research highlights a human resource challenge: persistent difficulties in providing incentives for healthcare providers to work in remote areas. These challenges extend beyond staffing issues and impact patients' access to care in rural regions, a key focus of the HSTP. Additionally, the same research added that the pace of change and digital advancements in the health system create difficulties for healthcare providers in securing funding and meeting improvement requirements, especially when infrastructure is not yet in place (8). These studies suggest that, although a roadmap has been outlined, challenges persist in the transformation process.

Moreover, research analyzing the evolution of public health policymaking in this evolving context remains limited (9, 10). Previous research has highlighted the need to better understand the role of policymakers in shaping health policies, underscoring a critical gap in the literature that this study seeks to address (11). Therefore, this paper aims to qualitatively examine the status of health policymaking in Saudi Arabia, including its formulation, development over the years, and the challenges encountered within the policymaking environment. These insights will be derived from a policy expert's perspective through semi-structured interviews. And since the VBHC strategy is a key driver of this transformation, researchers have focused on recruiting policy experts involved in it.

Finally, to place the analysis of the findings within the broader policy literature, the discussion will be guided by Kingdon's Multiple Stream Framework (MSF) (12), which explains how the three streams—policy problems, policy solutions, and political will—converge to create a “policy window” for reform. Using this framework will help understand the overall transition occurring in the Saudi health system as well as grasp the dynamics of health policymaking in Saudi Arabia.

## 2 Materials and methods

### 2.1 Study design

This study employed an exploratory qualitative research design using semi-structured key-informant interviews (KII) (13). This approach was chosen because research literature on policymaking in Saudi Arabia is scarce, and an in-depth understanding of the sector dynamics is needed.

### 2.2 Recruitment and sample

Since the VBHC strategy serves as a key driver of the health sector transformation, researchers have focused on recruiting policy experts involved in this strategy. To explore the status of health policymaking in Saudi Arabia, interviews were conducted with policy experts working in the entities forming the Value in Health (VIH) network (14). These experts are pivotal in shaping the policy landscape by translating VBHC principles into actionable reforms, and ensuring alignment with broader healthcare objectives. Their insights are crucial for understanding the decision-making processes, challenges, and facilitators influencing the policymaking process within the Saudi healthcare system. The VIH network comprises seven major entities representing the primary players driving the implementation of VBHC in the Saudi healthcare sector. Table 1 lists the seven entities and their role in the health sector.

**Table 1:** Value in health network entities and their role in the health sector.

**Entity**	**Type**	**Role**
Value in health center	Semi-government	National knowledge hub: VBHC policy, research, and education.
Council of health insurance (CHI)	Government regulator	Private health insurance regulator
National center for health insurance (CNHI)	Government agency	National health insurance payer
Saudi health council	Government council	Coordination, policy, research, data standards
Ministry of health	Government ministry	The primary regulator and provider of healthcare services
Public health authority	Government authority	Charged with protecting and promoting public health
Saudi commission for health specialties	Semi-government professional body	Licensing, accreditation, and training body

Using purposive sampling, we identified experts based on their direct involvement and roles in shaping health policy within their relevant organizations in Saudi Arabia. Experts were approached via LinkedIn messages. Inclusion criteria required experts to work in one of the entities within the VIH network, have direct experience in developing or implementing health policies, and have at least 5 years of experience in their organization. This ensures their complete involvement and thorough understanding of how policy is developed in Saudi Arabia. Although our sample is limited to the VIH network, these entities are central to policy development in Saudi Arabia. Future research could expand to include regulatory agencies and independent policy advisors to ensure a broader perspective. A snowball sampling technique was employed to expand the participant pool by identifying additional key experts recommended by interviewees. Table 2 provides a detailed landscape of the experts.

**Table 2:** A breakdown of the expert information.

**Expert**	**Entity**	**Current role**	**Background**	**Years of experience**
Expert 1	Entity 1	Health policy manager	Medical	10
Expert 2	Entity 2	Health policy expert	Medical	18
Expert 3	Entity 3	Health policy expert	Healthcare Management	17
Expert 4	Entity 4	Health policy director	Medical	9
Expert 5	Entity 2	Health policy manager	Medical	19
Expert 6	Entity 5	Health policy expert	Medical	27
Expert 7	Entity 6	Consultant	Medical	17
Expert 8	Entity 1	Health policy expert	Health economics	35
Expert 9	Entity 6	Advisor	Medical	32
Expert 10	Entity 4	Health policy manager	Medical	12

Regarding the sample size, recommendations differ on what constitutes an adequate sample size for interviews. A rule of thumb suggests that an adequate sample size in qualitative research can be determined through saturation (15). According to Hennink et al. (16), studies with homogenous populations and focused objectives reached saturation with 9–17 interviews; these two criteria were applied to the approach of this research, where experts share distinctive experiences and are asked questions that feed into defined objectives. Although experts have different professional backgrounds, their professional functions and organizational placement within the Value in Health Network were aligned. Also, they all had similar exposure to the national policy ecosystem under the HSTP. Thus, data saturation was determined through an iterative coding process. After the 10th interview, no new themes emerged, indicating thematic saturation, which is determined by the number of themes rather than the completeness of established theoretical categories (17).

Eighteen experts were contacted; five did not respond, three agreed but had scheduling conflicts, and since saturation was reached at 10, no follow-up communication occurred. Hence, the total number of experts was 10.

### 2.3 Instrument development

The interview guide was designed following the study objectives and a comprehensive review of the relevant literature mentioned in the introduction, incorporating open-ended questions to facilitate detailed responses. To ensure the guide remains relevant and clear, a subject matter expert reviewed it, and their feedback was incorporated into later revisions. Additionally, a pilot was conducted with a specialist in qualitative research methods. The research team conducted multiple iterations of the guide, refining the wording and structure of questions to enhance their comprehensibility and alignment with the study objectives. The guide was in English, as all experts preferred the interview in English. The guide is available in Appendix A.

### 2.4 Data collection procedures

Data were collected through semi-structured interviews conducted between June and August 2024. Interviews lasted 40–70 min and were either in-person or virtual, with the format chosen based on the expert's preference and availability. With the experts' written and verbal consent, all interviews were audio-recorded and transcribed verbatim. All interviews included the primary investigator (PI) and the expert only. No interviews were repeated. Notes were recorded after each interview for reflections and comments. Experts were free to request transcripts.

### 2.5 Data analysis

All transcripts were analyzed using inductive thematic analysis following Braun and Clarke's six-step framework (15). First, the analysis started with *data familiarization*, where transcripts were reviewed immediately after the interview, and notes and initial codes were generated; this was conducted after each interview. Second, *initial codes generalization*, where the codes were revised after each interview and amended accordingly. After the fourth interview, a codebook was created in Excel, revised by the research team, and iterative revision was done based on the team discussion. At this point, the analysis was moved to NVivo software (Version 15), which was used to facilitate data organization and coding; therefore, coding the rest of the transcripts was conducted using this software. Third, *themes identification*, where themes were inductively identified by deriving them directly from the data. Fourth was *themes revision*, where the research team revised themes and made changes until an agreement was reached. Fifth, *defining and naming themes* was carried out in a way that provided an answer to the objective of this paper. The final step, *producing the final report*, is being addressed in the results section. Additionally, while using Braun and Clarke's framework for coding and theme generation, the interpretation of the findings was guided by Kingdon's MSF, which describes how policy change happens when three independent streams—the *problem* stream (pressing issues), the *policy* stream (potential solutions), and the *political* stream (political will and drive)—come together to create a *policy window* for reform (12). Using this framework provided insight into how problem identification, solution recognition, and political motivation interacted to influence the Saudi health policymaking ecosystem.

### 2.6 Data management

Using inductive reasoning, notes and annotations were made in the transcript margins after the familiarization process to aid in coding and organization. This process was carefully aligned with the study objectives to maintain a clear focus. Additionally, the analysis was coordinated with data collection, so after each transcript was prepared, an analysis was performed on that specific transcript before moving on to the following interview. Many scholars recommended this approach as a way to ensure transparency and coherence (15, 18, 19).

The researchers used interpretation-focused or analytical coding, which is

“coding that comes from interpretations and reflection on the meaning.” (p. 206) (18)

This method involved revising the first transcript and breaking down the data into codes that offer meaningful insights related to the study's objective. These codes complement note-taking, annotations of reflections, and data interpretation. The researchers then identified similarities among these codes and grouped those that seemed linked, creating a list of groupings for the first transcript. For the second transcript, a similar grouping list was made and compared to the first. A third list was then created and compared with the previous two, with adjustments made as needed. This process continued until all transcripts were analyzed, resulting in a master list of major data groups that reflected subthemes in the transcripts. These subthemes were then grouped to form themes. The researchers conducted this process through regular meetings, revising the lists, and ultimately reached an agreement on the themes and subthemes. Appendix B provides a sample coding tree for the first theme.

## 3 Results

Three main themes emerged from the interviews: *theme one* concerns the *Historical Context and Evolution of Health policymaking, theme two* focuses on *Contemporary Drivers of Policymaking Development*, and *theme three* tackles the *Challenges in Policy Implementation*. Table 3 shows Emerging Themes and Sub-Themes from Data Analysis. Generally, the interviewees emphasized how policy development has evolved from outdated, “one-size-fits-all” models to more customized, population-specific approaches.

**Table 3:** Emerging themes and sub-themes from data analysis.

**Themes**	**Subthemes**
**Theme one: historical context and evolution of health policymaking**	Centralized approaches
	Reliance on foreign experts
	Vague policy sources
**Theme two: contemporary drivers of policymaking development**	Launch of health sector transformation program
	International collaboration and benchmarking
	Growing interest in evidence-based policy
	Encouragement of stakeholders engagement
	Investment in local capabilities
**Theme three: challenges in policy implementation**	Conceptual misunderstanding of policy
	Insufficient policy evaluation
	Lack of policy governance
	System fragmentation

### 3.1 Theme one: historical context and evolution of health policymaking

This theme provides the historical foundation and transformation of health policymaking in Saudi Arabia, shedding light on its centralized approach, reliance on foreign expertise, and the vague origin of many policies.

#### 3.1.1 Centralized approach

The experts emphasized that policymaking in Saudi Arabia was formulated using a centralized approach characterized by a top-down technique, as clearly stated by an expert,

“*the mechanism of policymaking where it was purely top-down*.” [Male, Entity 4]

And decisions were made at a senior level without much engagement from stakeholders working at the operational level. One of the experts explained the position of stakeholders to these policies, stating:

“*We were mainly reactive rather than proactive in policymaking*.” [Male, Entity 2]

#### 3.1.2 Reliance on foreign experts

Many experts have expressed concerns regarding the Saudi system's previous dependence on foreign expertise, as it provided technical knowledge without fully understanding the local context. In their view, this resulted in policies that did not align with the actual needs of the population. As stated by an expert,

“*We used to bring consultants who knew much about the issue at hand but at the same time knew so little about the context*.” [Female, Entity 3]

Another explained

“*Most people will look into the international practice and then replace it here; they would say that in the US, they implemented this policy, and it was a success, and therefore, we need to apply it here. This happens without looking into the need of the local context*.” [Male, Entity 2]

#### 3.1.3 Vague policy sources

Some experts expressed that the origin of health policies in the Saudi health system was unclear, and the rationale for implementing them was vague. One expert expressed their views indicating that:

“*Previously, I thought that the Saudi context was a one-size-fits-all situation. We used to use policies that have been around for decades, and no one knows where they came from... you don't know their origins or how did they start*.” [Female, Entity 1]

This uncertainty often results in misunderstandings of the aim of specific policies, which another interviewee highlighted.

“*We didn't understand the reason behind these policies that were published*.” [Female, Entity 5]

### 3.2 Theme two: contemporary drivers of policymaking development

Five key factors were identified as shaping the evolution of Saudi Arabia's health policymaking. These factors highlight the increasing emphasis on national capacity-building, evidence-based decision-making, and international collaboration, all aligning with Vision 2030.

#### 3.2.1 The launch of health sector transformation program (HSTP)

The HSTP has been instrumental in fostering a more adaptive and progressive approach to policymaking. Experts highlighted its effectiveness in customizing policies to meet local needs and supporting long-term planning. It was explicitly pointed out that the HSTP played a massive role in this development, as stated by one of the experts:

“*The evolution of health care and policymaking comes from the transformation that is happening in the kingdom*.” [Male, Entity 6]

The benefits of this program influenced the development of policies tailored to the population's needs. One expert mentioned that:

“*With the transition that is happening, we're seeing that policies can be more customized toward our population, our population needs*.” [Female, Entity 1]

#### 3.2.2 International collaboration and benchmarking

Global partnerships and comparative analysis are essential for advancing health policy development. Saudi Arabian policymakers actively collaborate with international organizations and benchmark against successful models from other countries. An expert pointed out one example of international collaboration: *We, for example, look at multiple international organizations. The OECD has an excellent and effective healthcare department that works on different policies and policy recommendations concerning health, and we try to engage with them as much as possible* [Female, Entity 1].

Another expert highlighted the advantage of the benchmark assessment in offering numerous policy options to decision-makers:

“*We go and see the benchmarking of the other countries and how they succeeded. After that, we will provide the policy options that align with our needs*.” [Female, Entity 3]

Learning from the best practices of other international organizations is increasingly evident, as noted by an expert:

“*You need to know what are the policies from other countries that are tackling this issue to understand what others are doing and learn from them, like the G20 and the OECD, which are the ones that we benchmark ourselves with regularly*.” [Male, Entity 2]

#### 3.2.3 Growing interest in evidence-based policy

A central theme that emerged during the interviews was the increasing reliance on evidence and data to inform health policies. Experts emphasized that evidence-based approaches have become vital for prioritizing and developing health policies. One expert highlighted the realized effect of evidence-based policies:

“*When we look at the health policies now, we can see that there has been an improvement and willingness to develop policies based on evidence and scientific research. Now, there is a global and local transformation in the sector where it shows that once you invest in the evidence, you will get good policies and good outcomes*.” [Male, Entity 2]

Another one emphasized,

“*I think what most of the entities have in common is they look at the evidence, they look at numbers, they look at data, and this is what should be focused on*.” [Female, Entity 1]

#### 3.2.4 Encouragement of stakeholders engagement

Experts emphasized the significance of engaging various stakeholders, including clinicians and the public, in the policymaking process to ensure improved policy design and commitment.

“*I would say there has been a comprehensive engagement with multiple stakeholders within different levels*.” [Male, Entity 6]

Moreover, stakeholder engagement has reached a new level, with concerned centers noting the impact of their engagement in adopting these policies once implemented. An expert highlighted their experience:

“*What we have noticed in the center is that the more you involve your stakeholders in the policy-making process, the more committed they become later when the policy gets enacted*.” [Male, Entity 5]

#### 3.2.5 Investment in local capabilities

A developing trend in Saudi health policymaking is fostering local expertise to reduce reliance on external advisors and strengthen the local experience. As observed by an expert:

“*I think one of the main elements of the enhancement in the policy-making process is that now we are starting to see local experts in the field of policymaking. We did not see that before*.” [Female, Entity 5]

### 3.3 Theme three: the implementation challenges facing policymakers

The analysis identified substantial obstacles that hinder the effective implementation of health policies in Saudi Arabia. These obstacles include conceptual misunderstanding of policy, insufficient policy evaluation, lack of governance, and system fragmentation.

#### 3.3.1 Conceptual misunderstanding of policy

Experts expressed a misunderstanding concerning the policies' clarity. This ambiguity hinders effective policy development and implementation. As pointed out by an expert,

“*I think it is crucial to mention here the misunderstanding and mix-up between policy, process, and procedure*.” [Male, Entity 3]

Another explained,

“*some of the policies do not have a clear objective, and it is vague and not precise; this will cause a challenge as you will not be able to measure its success or even evaluate its implementation as you do not know what exactly you want from this policy*.” [Male, Entity 2]

#### 3.3.2 Insufficient policy evaluation

The lack of systematic policy evaluation processes was a recurring theme, with experts highlighting the challenges of assessing the success and impact of implemented policies.

“*Something that I firmly believe was missing then and is missing now... is policy evaluation*.” [Male, Entity 1]

#### 3.3.3 Lack of policy governance

Governance issues have emerged as a significant barrier, with experts emphasizing the absence of coordinated leadership and comprehensive frameworks to steer policymaking and implementation. An expert shared the view:

“*We need more collaboration, to be honest. Everyone is trying to lead from their section, which is fine, but we need the governance*.” [Male, Entity 2]

#### 3.3.4 System fragmentation

Fragmentation in the healthcare system has been recognized as hindering cohesive policymaking efforts. Experts emphasized the necessity for better coordination and integration throughout the system.

“*There is the issue when looking from a health system perspective, the fragmentation in the healthcare system, which also brought to our attention the need for more catered health policies and more of health system thinking*.” [Female, Entity 1]

## 4 Discussion

Our study examined the health policymaking landscape in Saudi Arabia by investigating how health policies were formulated, the factors that influenced their development, and the challenges policymakers are facing. To interpret the findings, the discussion utilizes Kingdon's MSF with its three streams: problem, policy, and politics. This framework helps place the development of health policymaking in Saudi Arabia within the wider context of policymaking literature and clarifies why recent reforms have received considerable attention and had a notable impact.

Historically, the findings revealed that health policymaking in Saudi Arabia has been centralized, with minimal stakeholder engagement in the policy formulation process. From the experts' perspectives, this centralization was characterized by adapting health policies that heavily relied on foreign experts, who possessed technical knowledge but lacked adequate contextual adaptation. This, as a result, sometimes led to the introduction of policies that were not aligned with local needs. This observation aligns with evidence from other Gulf Cooperation Council (GCC) countries. For example, the United Arab Emirates (UAE) and Qatar, which have also begun their health transformation journeys, still face structural challenges rooted in the old health system where reliance on imported models created gaps between policy design and implementation (20). Unlike the Saudi approach, which is solely centralized and directed by the government through the MOH, the UAE's federal system incorporates both centralization and decentralization. Decentralization is evident in the emirate-level flexibility and autonomy, allowing for more localized policies. Simultaneously, national-level centralization can be enacted when necessary. For instance, before 2025, individual emirates had the autonomy to offer health insurance to their citizens and expatriates. However, by January 2025, it became mandatory for all emirates to provide health insurance to everyone (21).

Beyond the GCC, some countries have a history of switching between the two approaches, depending on their health system needs, as in Brazil and Spain. In these countries, efforts are now underway to recentralize to enhance health system efficiency and coordination, which were affected by decentralization (22). Scholars in the field refer to this as a complex debate, with each approach offering advantages and challenges. Centralization can be beneficial for establishing nationwide policies because it streamlines decision-making and ensures uniformity across regions, while allowing for greater control and coordination. However, this approach frequently increases bureaucracy while decreasing responsiveness to local needs (22). Meanwhile, decentralization enhances responsiveness and accountability, although it may result in higher spending (23). In both approaches, the main challenge, and the key to success if managed correctly, is balancing centralized authority with active stakeholder involvement to ensure policies are both technically sound and locally relevant.

The literature notes that economic development and political change significantly influence health policymaking by shaping the priorities, resources, and frameworks within which policies are developed and implemented (1). This is exemplified by Saudi Arabia's Vision 2030 and the HSTP, which have collectively reshaped the health policy landscape. Achieving Vision 2030 strategic objectives required a fundamental shift in decision-makers mindset, empowering them to remain responsive to evolving healthcare demands and align their efforts accordingly—a perspective echoed by policy experts interviewed in this study.

Additionally, the growing interest in evidence-based policy that is driven by data and research, as highlighted by experts, is a positive trend that aligns with global patterns and is supported by the recently published Saudi Health Council manual, which serves as a guide for policymakers, emphasizing the role of evidence-based policy in the policymaking process (24). Global interest in evidence-based policies has gained popularity over the last 20 years, particularly in response to the increasing challenges facing the health industry worldwide (25). Researchers argue that evidence-based policy offers a solution to these challenges, as it helps prioritize cost-effective and beneficial interventions, thereby reducing wasteful spending on ineffective ones (26). However, there has been a recent shift from evidence-based policies to evidence-informed policies, where researchers argue that evidence-based policies often focus only on certain types of evidence without considering contextual factors. Therefore, the move toward evidence-informed policies, they suggest, allows for the inclusion of rigorous research alongside other elements (27). With that being said, both approaches present a challenge in the Saudi context, especially since empirical research indicates that Saudi literature lacks studies evaluating the progress of reform following HSTP, which in turn hinders efforts to inform policies (5–7). Therefore, this national commitment to evidence-based practice should accelerate efforts to enhance data capture and dissemination, allowing for intersectoral collaboration and research.

This evolution of health policymaking in Saudi Arabia can be understood through Kingdon's MSF, which suggests that significant policy change occurs when the problem, policy, and political streams align (12). In this context, the problem stream reflects the long-standing structural and operational challenges that have consistently pressured the Saudi health system, as mentioned earlier in the findings and introduction. The policy stream shows a growing readiness for solutions, illustrated by the system's increasing move toward evidence-based policymaking, the use of benchmarking as a learning tool rather than just imitation, and investments in developing local expertise and stakeholder engagement. Altogether, these developments indicate the system's preparedness to enter a new phase in its policymaking environment, supported by the growing availability of international models and evidence-based practices that focus on value and efficiency. The political stream emerged from the government's strong commitment to reform under Vision 2030, and a clear example of this political influence is the elevation of VBHC as the main strategy through top-down agenda-setting, driven by the HSTP. The alignment of these three streams created a distinct policy window—represented by Vision 2030 and the HSTP—which facilitated the quick implementation of transformational health reforms. This explains the rapid policy changes observed in the Saudi context and the key role of high-level political commitment in shaping the reform agenda—a pattern also seen in other GCC income countries undergoing national transformations (21).

Nevertheless, while this transformation represents a significant and positive step, it is essential to consider its possible limitations carefully. With the recent transformation in the country and the effort to empower local policymaking capabilities, experts have observed a conceptual misunderstanding of policy among stakeholders, leading to policies that are neither clear nor well understood. This conceptual ambiguity leads to delays and challenges in implementing and complying with policies. In the UK, for example, this ambiguity in policy statements and goals led to significant tension among stakeholders during the implementation of the New Care Model due to the confusion between priorities and expectations, which made it difficult for stakeholders to align their efforts effectively (28). A recent study in Saudi Arabia examined the conceptualization of VBHC policies and identified that there were inconsistencies in interpreting what VBHC entails and how it should be implemented. This inconsistency stemmed from a lack of clarity in how the strategy was introduced and communicated (11). These issues have been documented in comparative health policy studies, where varied interpretations among policymakers, administrators, and practitioners resulted in fragmented implementation (4).

On a technical level, experts agree that although policy evaluation is an acknowledged step in the policymaking cycle, the health system in Saudi Arabia, in practice, lacks this step. Poor policy evaluation creates issues in assessing the effectiveness of implemented policies and determining whether they achieved their intended goals. Policy evaluation is lacking globally, with only 27 out of 109 countries having formalized policy evaluation frameworks, and even among these 27 cases, the use of these evaluation frameworks is not consistent, resulting in the development of ineffective health policies (29). A report published by the Global Parliamentarians Forum for Evaluation (GPFE) provided an overview of national evaluation policies across various countries. One of the report's main findings is that contextual factors, such as the country's governmental structure, legislative support, external influences, and institutional capacity to perform the evaluation, played a significant role in implementing policy evaluation. While some countries have succeeded in this area, such as Finland, Germany, and Ghana, others are still struggling to achieve a satisfactory level (30). Therefore, collaboration between policymakers and researchers should be strengthened in Saudi Arabia to facilitate the initiation of policy evaluation, which could later be institutionalized through enhanced policy governance. The limited policy evaluation in Saudi Arabia may result from the long history of centralized policymaking, in which policies were often enacted without strong ties to population needs. This has created a culture where policies are followed instead of questioned, and evaluation is often seen as a form of inspection or criticism rather than a chance to learn (31). These patterns show how a strong political stream, through control and rapid reforms, has overshadowed the development of organized evaluation systems, leading to habits that prioritize compliance over reflection and improvement.

Lastly, the findings highlighted a critical barrier to policy success: governance gaps that lead to system fragmentation. System governance is defined as

“a process whereby societies, institutions, and organizations make important decisions, determine how they are involved in the process and how they render an account.” (32)

Weak governance structures contribute to systemic inefficiencies, reduced transparency, and a lack of alignment in policymaking processes, especially between policy design and implementation. Without a clear governance framework, stakeholders operate based on individual interpretations and priorities, leading to fragmented efforts and inconsistent policy implementation (33). Many countries face similar governance challenges that hinder effective policy implementation. For instance, a qualitative study in Ireland revealed that weak governance, characterized by issues with accountability, a lack of transparency, and ambiguous roles and responsibilities, hindered the successful implementation of health reforms (33). A study in South Korea found that the lack of effective governance in the new payment system, combined with a top-down approach and limited political cooperation, negatively impacted the health system and led to unintended consequences (34). In contrast, countries like Canada have established robust governance frameworks, demonstrating how well-structured policy systems contribute to improved overall performance and a more efficient health system (35). Similar to the issue of limited policy evaluation, the continued presence of weak governance can be linked to the highly centralized authority structure, where policymaking stays within ministerial boundaries rather than being coordinated across sectors. This centralization limits opportunities for shared learning and accountability. Similar patterns have been seen in other GCC countries, where hierarchical systems often hinder collaborative governance (21, 36).

In sum, although experts often highlight positive reforms under Vision 2030 and the HSTP, it is important to recognize that such large-scale reforms can also have unintended consequences. For instance, rapid restructuring might cause transitional gaps in service delivery, while increased reliance on international benchmarking could neglect locally specific needs. Similarly, the intense political drive to deliver quick results fostered a pace of reform that frequently surpassed the necessary development of robust governance, effective evaluation frameworks, and clear accountability mechanisms. Additionally, although evidence-based approaches are praised, their successful implementation depends on data quality and governance structures, which remain underdeveloped. Additionally, the speed and scale of the transformation make it difficult to identify the true success factors; some improvements in the health sector could result from reforms in other sectors, making it hard to establish a clear causal relationship about what exactly caused these improvements or failures. These critical considerations demonstrate that reforms should be viewed as dynamic processes with both opportunities and risks, rather than universally positive changes.

These insights emphasize crucial aspects that require focus to enhance the efficiency of health policymaking in Saudi Arabia. Clearly defining policy concepts, creating robust evaluation frameworks, tackling governance deficiencies, and minimizing systemic fragmentation are essential measures for developing unified and effective health policies.

### 4.1 Implications for policy and practice

This study highlights the critical need to strengthen local policy capacity in Saudi Arabia to reduce reliance on foreign expertise, which, while valuable, often lacks the contextual grounding necessary to address local health system needs. Developing national expertise in should therefore be a strategic priority, achieved through the establishment of dedicated policy think tanks, academic–industry partnerships, and structured training programs that equip professionals to lead policy formulation and evaluation. Incorporating health policy education into medical and health professional curricula would also enhance understanding of policy fundamentals and foster a new generation of policymakers.

At the same time, systemic improvements are essential to ensure that reforms achieve their intended outcomes. Greater clarity in policy communication, robust evaluation frameworks, and stronger governance mechanisms are needed to translate reform ambitions into sustainable change. Establishing a centralized digital repository to document policy development and rationale would improve transparency, while investing in a national health data ecosystem would support responsive, evidence-informed decision-making that remains sensitive to the Saudi context.

### 4.2 Limitations and future research

This study has its limitations. One key limitation is the reliance on qualitative interviews of policy experts, which, although rich in contextual insights, may capture subjective viewpoints that do not fully represent the broader range of experiences across the Saudi healthcare system. Future research could employ a mixed-methods approach, integrating policy documents, quantitative surveys, or longitudinal data to validate and build upon the themes identified here, as well as including other stakeholders such as healthcare providers and patients. Secondly, this study is exploratory by nature, relying on a purposive sample of policy experts from the Value in Health network. While these experts provided valuable insider perspectives, the sample size limits the ability to represent the full spectrum of policymakers in Saudi Arabia and reduces the generalizability. Therefore, the findings should not be interpreted as broadly representative or causal. Instead, they offer preliminary insights into policymaking processes that require further investigation and triangulation using larger, more diverse methods. Thirdly, this research primarily addressed the historical and structural elements of health policymaking in Saudi Arabia, with less focus on the outcomes of these policies. Further studies could examine the real-world impacts of policy transitions, assessing their effectiveness and identifying gaps between policy intentions and practical outcomes. Finally, future research is needed to explore the broader social and political factors that influence policy dynamics.

## 5 Conclusion

In conclusion, while Saudi Arabia's health reforms reflect a strong ambition toward inclusivity, evidence-informed decision-making, and multi-sectoral collaboration, these aspirations continue to face significant obstacles. Conceptual ambiguity, limited evaluation mechanisms, and fragmented governance remain interrelated barriers that undermine effective implementation—unclear policy ideas prevent shared understanding, weak evaluation hinders learning from past efforts, and governance gaps leave stakeholders working in silos. The contrast between a historically centralized system and current aspirations for more collaborative, evidence-driven policymaking underscores the transitional and contested nature of health policy in the Kingdom. Although reforms are often presented as progressive, the pace of restructuring and reliance on international benchmarks risk overlooking local realities. Evidence-based approaches can provide valuable tools for efficiency and accountability, but their success ultimately depends on strengthening data infrastructure and institutional capacity, both of which remain underdeveloped. Finally, it is important to acknowledge that the conclusions of this study are exploratory and offer initial insights into policymaking practices in Saudi Arabia. Therefore, the findings serve as a foundation for further research to validate and expand upon these themes.

## Data Availability

The original contributions presented in the study are included in the article/Supplementary material, further inquiries can be directed to the corresponding author.
