# Supplementary material for: Tracing the evolution of health policymaking in Saudi Arabia: a qualitative analysis of experts' opinion
Source: Front Public Health. 2026 Jan 22;13:1672410. doi: 10.3389/fpubh.2025.1672410 (PMC12872759; doi:10.3389/fpubh.2025.1672410)
Supplement: Supplementary file 1 [file Table_1.docx]

Supplementary Material

**Appendices**

Appendix A: Interview guide.

1. What sparked your interest in health policy, and how did you become involved, particularly in the context of Saudi Arabia?
2. Could you share details about your background and current role in Saudi Arabia's health policy?
3. How do you perceive the evolution of policymaking in Saudi Arabia, especially in healthcare? Can you describe your contribution to this process?
4. Can you outline the process of health policy formulation in Saudi Arabia?
5. Based on your experience, do you believe the policy formulation process is consistent across different health policies?
6. What are the main challenges and obstacles you encounter as a policymaker when developing and implementing health policies?

Appendix B: Theme One Sample Coding Tree

| **Theme One: Historical Context and Evolution of Health Policymaking** | |
| --- | --- |
| **Subtheme 1.1: Centralized approaches** | |
| **Category: nature of policymaking** | **Code:** top-down – ‘*the mechanism of policymaking where it was purely top-down’*  **Code:** high-level – ‘*we have some policies shaped on a higher level’*  **Code:** mandated **– ‘***policies were mandated’* |
| **Subtheme 1.2: Reliance on Foreign Experts** | |
| **Category: who makes policies** | **Code:** international experts – ‘*Sometimes, it will be expat experts who are missing local data’*  **Code:** limited expertise – ‘*usually it develops within certain expertise, not necessarily to have any deep analysis in terms of understanding the context’*  **Code:** consultant – ‘*consultants who knew much about the issue at hand but at the same time knew so little about the context.’* |
| **Category: lacks contextual knowledge** | **Code:** no local data – ‘*Sometimes, it will be expat experts who are missing local data’*  **Code:** doesn’t understand local context – ‘*usually it develops within certain expertise, not necessarily to have any deep analysis in terms of understanding the context’*  **Code:** little contextual knowledge – ‘*consultants who knew much about the issue at hand but at the same time knew so little about the context.’* |
| **Subtheme 1.3: Vague Policy Sources** | |
| Category | **Code:** unknown policy origin – ‘*no one knows where they came from ...* *you don’t know their origins or how did they start’*  ***Code:*** no understandable logic *- ‘We didn’t understand the reason behind these policies that were published’* |
